# Digital Community Inclusion of Individuals With Serious Mental Illness: A National Survey to Map Digital Technology Use and Community Participation Patterns in the Digital Era

**DOI:** 10.2196/28123

**Published:** 2021-09-21

**Authors:** Carmit Noa Shpigelman, Amir Tal, Yaara Zisman-Ilani

**Affiliations:** 1 Department of Community Mental Health Faculty of Social Welfare and Health Sciences University of Haifa Haifa Israel; 2 Beit Ekstein Danel Group Haifa Israel; 3 Department of Social and Behavioral Sciences College of Public Health Temple University Philadelphia, PA United States

**Keywords:** mobile health, technology, digital community participation, digital community inclusion, serious mental illness, recovery

## Abstract

**Background:**

Despite the growing interest in developing and using mobile health (mHealth) and digital technologies in mental health, little is known about the scope and nature of virtual community inclusion.

**Objective:**

The overarching goal of this study was to understand and conceptualize virtual community inclusion of individuals with serious mental illness (SMI). Specific objectives of this study were as follows: (1) mapping the prevalence, trends, and experiences related to mHealth and digital technology use among individuals with SMI; (2) comparing patterns of technology use by individuals with and those without SMI; and (3) examining whether use of mHealth and digital technologies predicts recovery among individuals with SMI.

**Methods:**

A web-based survey of technology use and virtual participation was developed and distributed among adults with and those without SMI via social media, national email discussion lists, nonprofit organizations, and advocacy groups.

**Results:**

A total of 381 adults aged 18 years or older participated in the survey, of whom 199 (52%) identified as having a SMI. Participants with SMI reported significantly greater access to technology and significantly fewer days of face-to-face participation in community activities than those without SMI. Among participants with SMI, greater technology use was positively associated with positive emotions and significantly predicted recovery.

**Conclusions:**

This study is the first to explore, map, and conceptualize virtual community inclusion among adults with SMI. Our findings indicate a gap in the literature and research on community inclusion and participation, and emphasize the need for virtual community inclusion, particularly during the COVID-19 pandemic and its future implications.

## Introduction

The COVID-19 pandemic has emphasized the necessity of mobile health (mHealth) and the use of digital technology more generally in managing chronic health conditions [1-4]. Recently, the World Health Organization has defined mHealth as the use of mobile and wireless technologies to support the achievement of health objectives [5]. In the field of mental health, digital technologies have been integrated for various purposes, including research, intervention development, diagnosis, and prevention [6-9]. Individuals with serious mental illness (SMI) may benefit from mHealth interventions to learn where to seek help, cope with stigma, access mental health services, and use digital technologies to coordinate among various service providers, especially in peripheral areas [1,10-13].

A pioneering survey conducted in the United States among individuals who self-identified with schizophrenia [14] indicated that 90% owned more than 1 internet-connected device, particularly smartphones, and frequently used digital technologies. A meta-analysis [8] that assessed mobile phone ownership as well as interest in mHealth among individuals with psychosis found that approximately 60% were interested in using novel smartphone apps for monitoring their mental health status. Another systematic review [15] indicated a high rate of adherence to mobile technologies (83%) among people with SMI.

Recently, studies that developed and examined mHealth interventions for individuals with SMI have confirmed the feasibility and acceptability of these emerging interventions [1,15-18]. These studies also provide preliminary support for the notion that individuals with SMI can benefit from mHealth interventions [19-21]. In addition, research has indicated that social use of digital technologies is associated with community participation, which can be valuable for individuals with SMI [22].

Community participation is a multidimensional concept defined as “active involvement in activities that are intrinsically social, and either occur outside of the home or are part of a non-domestic role, such as work, social (outside of the household), and other community roles” [23-25]. Community participation behaviors include involvement in recreational, social, vocational, civic, and other areas of community life, and have been found to contribute to the recovery process and quality of life of individuals with SMI [26-28]. A dynamic approach to recovery was applied in the context of the present study. Recovery (also known as “recovery in”) refers to a subjective process characterized by movement toward conditions of hope, purpose, and wellness. This concept of recovery emphasizes the person’s self-determination and participation in life pursuits as education, employment, friendship, and spirituality, consistent with his/her goals, values, and preferences [29,30]. In this sense, using digital technologies can contribute to greater involvement in physical and web-based activities and consequently to the individual’s recovery.

Use of digital technologies and mHealth interventions may be particularly valuable in the current global pandemic. The COVID-19 crisis has posed significant challenges for the delivery of mental health services [31]. Policies of quarantines and social distancing [32] have forced many practitioners to adjust quickly to using digital technologies [33-36]. This unprecedented crisis presents an imperative for mental health care systems to make mHealth interventions available as a routine part of care. However, there is lack of information about differences in digital participation between individuals with and those without SMI in routine care. Although there is growing literature on face-to-face community participation of individuals with and those without SMI [37], less is known about participation in the digital community among individuals with SMI. In this study, we conceptualize digital participation to describe involvement in social activities and roles within the digital space, such as recreational, social, vocational, civic, and other areas of community life. Knowledge and use of digital technologies are crucial for digital participation; however, to date, this field of research has been limited. In addition, the association between mHealth and the use of digital technologies and recovery of individuals with SMI should be further explored. Comparative data about virtual participation in routine care are valuable in identifying additional pathways for recovery, especially under the current conditions where human communication is so predominantly internet-based.

This study addresses these gaps by exploring patterns of digital participation among individuals with and those without SMI. Our specific objectives were (1) mapping the prevalence, trends, and experiences related to mHealth and digital technology use among individuals with SMI; (2) comparing the usage patterns of individuals with and those without SMI; and (3) examining whether the use of mHealth and digital technologies predicts recovery among individuals with SMI.

## Methods

### Setting and Survey Development

A web-based survey of technology use and participation was developed for dissemination in Israel on the basis of the National Alliance on Mental Illness (NAMI) mHealth survey [14]. The original survey was translated to Hebrew, adapted to the local context, and supplemented with additional items pertaining to recovery [14] and community participation [37]. The final version of the survey included four sections: (1) technology use, (2) recovery, (3) community participation, and (4) background and demographic characteristics.

### Measures

#### Technology Use

Survey questions focused on access to digital devices (eg, laptop or smartphone), frequency of use, purposes of use (eg, contacting friends, family, and psychiatrists), emotional experience while using digital technologies (negative or positive emotions), and helpfulness of activities using the devices in managing mental health (only for respondents with SMI). Survey questions were translated to Hebrew and adapted from the 2014 NAMI mHealth survey [14].

#### Recovery

The Recovery Assessment Scale is a valid measure of recovery in mental health research. It was originally developed as a 41-item measure by mental health consumers through an analysis of recovery stories that resulted in the identification of 39 themes of the subjective experience of recovery [38]. In this study, we used the shorter 12-item Recovery Assessment Scale with a 1-5 response scale and Cronbach *α* values ranging .60-.97 [39,40]. For this study, Cronbach *α*=.87.

#### Community Participation

This variable was assessed using the community participation measure [41], a self-report instrument examining the amount, sufficiency, and importance of participation in 26 different areas of community-based activities over the previous 30 days. Individuals are asked to report the number of days that they participated in each activity without a staff member (amount), whether their level of participation was “enough,” “not enough,” or “too much” (sufficiency), and whether the activities were important to them. We extracted the following to serve as independent variables in our analyses: *amount* of participation, defined as total participation days across all items (range 0-780 [30 days × 26 participation areas]); and *extent* of participation (referred also to as diversity of participation in other research, n=41), defined as the number of unique participation areas (ie, items) with at least 1 day of participation reported. For this analysis, Cronbach *α*=.93.

### Procedure and Analysis

The survey was distributed in Israel between February 2018 and May 2019 among adult individuals (over the age of 18 years) who identified as having SMI (the study group) and those without SMI (a comparison group), based on a screening question. SMI was defined on the basis of the Substance Abuse and Mental Health Services Administration criteria [42], which are acceptable for distribution of block grants to state governments, and the Israel National Insurance Institute criteria in Israel for disability support [43,44]. Accordingly, individuals with chronic conditions, such as schizophrenia, major depression, or another mental health disorder that results in serious disability are considered under the umbrella of SMI [45,46].

Before large-scale dissemination, we conducted a field-test of the final version of the survey with 4 individuals with SMI who commented on the questions and wording and further refinement. Survey participants were recruited via a snowball approach using Facebook groups and national email discussion lists (“Listservs”) of consumers and service providers as well as via nonprofit organizations and advocacy groups of and for people with SMI. We also provided an option of a paper-and-pencil version of the survey for individuals with SMI who were interested in participating via a face-to-face meeting with the research assistant.

The study was approved by both the University of Haifa Institutional Review Board and Israel Ministry of Health. SPSS (version 25, IBM Corp) was used to present descriptive statistics and calculate correlations and means differences. A *P* value of <.05 was set as the significance level.

## Results

### Participants

The sample includes 381 respondents, of whom 199 (52%) reported having at least 1 SMI, with 16 (8%) having reported more than 1 SMI. The most common condition was schizophrenia (n=58, 29.1%), followed by affective disorders (bipolar disorder, n=16, 8%; depression, n=12, 6%; and anxiety n=5, 2.5%), personality disorders (n=7, 3.5%), and posttraumatic stress disorder (n=6, 3%). Most respondents with SMI (n=157, 78.9%) reported an additional nonmental health chronic condition, mostly diabetes (n=10, 6.4%) or an orthopedic condition (n=10, 6.4%), compared to those without SMI, who reported no such condition.

More women (n=246, 64.6%) than men were included in the survey, although the distribution was more balanced among those with SMI (Table 1). The most frequent age category was 18-34 years (n=146, 38.3%). While most respondents with SMI were single (n=129, 64.8%), the majority of respondents without SMI were married (n=103, 56.6%). While most of the respondents without SMI were employed (n=131, 72.0%), most respondents with SMI were employed only in supported employment programs (n=106, 53.3%). Lastly, while most respondents with SMI had at least a high school diploma (n=80, 40.2%), the majority of respondents without SMI had a higher education degree (n=136, 74.7%).

**Table 1:** Participant characteristics (N=381).

Variables		Entire sample, n (%)	Respondents with SMI^a^ (n=199, 52.2%), n (%)	Respondents without SMI (n=182, 47.8%), n (%)	*P* value	
**Gender**						<.001
	Female	246 (64.6)	109 (54.8)	137 (75.3)		
	Male	135 (35.4)	90 (45.2)	45 (24.7)		
**Age (years)**						.06
	18-34	146 (38.3)	72 (36.2)	74 (40.7)		
	35-46	106 (27.8)	65 (32.7)	41 (38.7)		
	47-64	103 (27.0)	53 (26.6)	50 (27.5)		
	>65	26 (6.8)	9 (4.5)	17 (9.3)		
**Marital status**						<.001
	Single	185 (48.6)	129 (64.8)	56 (30.8)		
	Married	134 (35.2)	31 (15.6)	103 (56.6)		
	Separated/Divorced/Widowed	62 (16.3)	39 (19.6)	23 (12.6)		
**Religion/ethnicity**						.04
	Jewish	356 (93.4)	187 (94.0)	169 (92.9)		
	Muslim	9 (2.4)	1 (0.5)	8 (4.4)		
	Christian	4 (1.0)	2 (1.0)	2 (1.1)		
	Other	12 (3.1)	9 (4.5)	3 (1.6)		
**Housing status**						<.001
	Alone	74 (19.5)	55 (27.8)	19 (10.5)		
	With a partner	121 (31.9)	33 (16.7)	88 (48.6)		
	With family	124 (32.7)	54 (27.3)	70 (38.5)		
	Supported housing	60 (15.8)	56 (28.3)	4 (2.2)		
**Employment status**						<.001
	Employed	183 (48.0)	52 (26.1)	131 (72.0)		
	Self-employed	24 (6.3)	7 (3.5)	17 (9.3)		
	Unemployed	48 (12.6)	31 (15.6)	17 (9.3)		
	Retired	19 (5.0)	3 (1.5)	16 (8.8)		
	Supported employment	107 (28.1)	106 (53.3)	1 (0.5)		
**Education level**						<.001
	Less than a high school diploma	30 (7.9)	27 (13.6)	3 (1.6)		
	High school diploma	105 (27.6)	80 (40.2)	25 (13.7)		
	Some college, no degree	37 (9.7)	19 (9.5)	18 (9.9)		
	Bachelor’s degree or higher	209 (54.9)	73 (36.7)	136 (74.7)		

^a^SMI: Serious mental illness.

### Comparing Male and Female Respondents

Overall, men reported having significantly greater access to technology (mean 3.58, SD 1.47) than did women (mean 3.01, SD 1.43; *t_379_*=3.62; *P*<.001). Women reported significantly greater negative emotions (mean 2.06, SD 0.86) when using digital technologies compared to men (mean 1.79, SD 0.83; *t_379_*=–2.95; *P*<.01). Compared to men, women also reported significantly more total days of community participation in terms of both amount (women: mean 42.02, SD 11.73; men: mean 37.67, SD 12.17; *t_312_*=–3.09; *P*<.01) and extent of participation (women: mean 20.02, SD 10.11; men: mean 17.30, SD 10.74; *t_262.3_*=–2.40; *P*<.05).

### Comparing Respondents With and Those Without SMI

Respondents with SMI reported significantly greater access to technology (mean 3.63, SD 1.43) than those without SMI (mean 2.57, SD 1.38; *t_379_*=–6.15; *P*<.001). Respondents with SMI also reported significantly greater negative (mean 2.05, SD 0.87) and positive emotions (mean 2.99, SD 0.96) when using digital technologies compared to those without SMI (negative emotions: mean 1.87, SD 0.80; *t_379_*=–2.06; *P*<.01; positive: mean 2.59, SD 0.78; *t_373.8_*=–4.43; *P*<.05). Finally, respondents with SMI reported significantly fewer days of participation in community activities (mean 37.6, SD 11.41) compared to those without SMI (mean 43.9, SD 11.92; *t_312_*=4.79; *P*<.001).

### Predicting Recovery Among Respondents With SMI

Multiple linear regression analysis was conducted to predict recovery among the respondents with SMI (n=199) based on their access to digital devices, emotional experience with technology use (negative and positive), and the amount and extent of their community participation. A significant regression model was obtained (*F*_5,163_=9.39; *R^2^*=0.224; *P*<.001). Greater experience of emotions while using technology, both negative (*β*=–.290; *P*<.001) and positive (*β*=.299; *P*<.001), and greater amount of community participation (*β*=.249; *P*<.01) significantly predicted recovery. Greater positive emotions and more days of community participation were positively related to recovery, while greater negative emotions were negatively related to recovery.

### Positive and Negative Emotions as Mediators of Recovery

**Figure 1:**
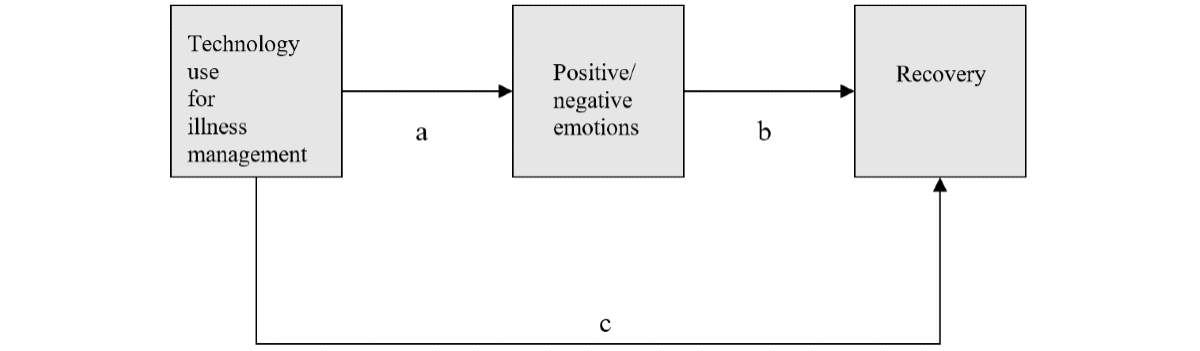
Mediation model.

Regression analyses were conducted to assess the contribution of technology use (for illness management) and emotions experienced while using the technology to recovery among respondents with SMI (n=199). First, we assessed the direct association between technology use for illness management (also referred to “helpfulness of activities when using technological devices” in the NAMI study [14] and recovery; Figure 1, path c). Significant and positive *β* weights were obtained (*β*=.19; *P*<.01).

Second, 2 regression analyses were carried out with technology use for illness management as the independent variable; 1 model included negative emotions and the other included positive emotions as the dependent variables (Figure 1, path a). Significant and positive *β* weights were obtained for both negative (*β*=.15; *P*<.05) and positive emotions (*β*=.50; *P*<.001).

Third, 2 multiple regression analyses were conducted to predict recovery by positive and negative emotions and technology use for illness management, respectively (Figure 1, path b). A negative and significant *β* weight was obtained between negative emotions and recovery when controlling for technology use (*β*=–.17; *P*<.05). A positive and significant *β* weight was obtained between positive emotions and recovery when controlling for technology use (*β*=.36; *P*<.001). A positive and significant *β* weight was obtained when predicting recovery with technology use as the predictor and controlling for negative emotions (*β*=.22; *P*<.01). No such result was obtained when predicting recovery with technology use as the predictor and controlling for positive emotions (*β*=.01; *P*>.05) (Figure 1, path c). These patterns suggest partial mediation between technology use (for illness management) and recovery when negative emotions serve as a mediator, and complete mediation in the case of positive emotions under the 4 conditions of mediation [39].

## Discussion

### Principal Findings

This study is the first to explore and conceptualize digital community inclusion among individuals with SMI. Our study focused on digital participation of adults with SMI in Israel. We compared the mHealth and digital technology use (ie, virtual participation) of adults with and those without SMI and explored whether and how participation factors predict recovery among adults with SMI. The main findings included higher rates of digital participation among individuals with SMI compared to those without SMI. In addition, greater positive emotions were noted when digital participation in the community, digital technology use, and more days of face-to-face community participation were positively related to recovery among adults with SMI. Positive emotions during digital community participation mediated the relationship between technology use (for illness management) and recovery. The findings emphasize the important role of digital participation on community inclusion of adults with SMI.

Previous studies that compared digital technology use among individuals with and those without SMI have yielded contradictory findings. On one hand, lower rates of technology access and use were observed among individuals with SMI than among those from the general population [47,48]. On the other hand, some studies found that individuals with SMI used digital technologies at rates similar to those of the general population, and concluded that mental health problems may not be a barrier to technology use [14,49,50]. Although the aforementioned studies found similar or lower rates of technology use among individuals with SMI, our study reported a higher rate of technology use among individuals with SMI than in the general adult population. A plausible explanation for this finding is that the other findings of this study showed that adults with SMI reported significantly fewer days of participation in community activities than those without SMI. Another plausible explanation for this finding is the growing number of mental health and wellness apps available for individuals with SMI [51,52]. Furthermore, using digital technologies for mental health care has created a more accessible environment for people with SMI, thus enabling anonymous participation. In case they decide to disclose personal information, nonverbal and non–face-to-face communication may create a less stigmatic environment for interaction with others [8,53,54].

In addition to exploring mHealth and digital technology use among adults with SMI, this study examined whether digital community participation predicted recovery. The theoretical framework of community participation traditionally refers to face-to-face or actual participation and is defined as the empowered, self-determined choice and action among individuals to be active in valued roles in the communities of their choice [26]. According to this traditional framework, the term “community participation” includes 3 main types of participation: *social* (eg, attending a community event, entertaining family or friends at home, or visiting family or friends), *productive* (going to school to earn a degree or certificate, working for pay, and participating in volunteer activities), and *leisure* (going to a museum, theater or cultural event, going to a park or recreating center, and going to a restaurant) [41]. Engaging with others in the community may also reduce public stigma toward people with SMI, which in turn can contribute to their recovery [55-57].

However, it seems that the life domains of community participation have referred to the physical environment, while participation in the digital environment has been excluded, although the digital environment has become an integral part of our life [58,59] and even more so in the COVID-19 era [4]. Following the concept of recovery as a dynamic process [30], studies have indicated that social support plays a main role in an individual’s recovery [60,61]. Social support can be delivered not only through face-to-face interactions, as demonstrated in the traditional concept of community participation and from a distance through remote communication using digital technologies [62]. While some preliminary studies focused on the impact of social media use on face-to-face community participation among individuals with SMI [22,63,64], they have focused solely on social media and did not include mHealth and digital technologies for illness management.

Moreover, recent studies have not conceptually included digital participation as part of community participation. This study has addressed this concept and theoretical gaps by exploring the predictive factors to recovery while taking into account both digital and face-to-face, in-person participation. As reported by Hendryx et al [65], involvement in a wide range of activities, whether they are more or less social in nature, physically active, or occur inside or outside of home, was related to better recovery. Hence, using digital technologies for greater involvement in physical or digital activities, whether the activities are intended for spending time alone or for contacting others socially, can empower people to manage their recovery [66]. Our findings provide further support to this argument by showing that using technology for illness management predicted recovery.

Furthermore, emotional experience of technology use were found to mediate the relationship between technology use and recovery, while positive emotions completely mediated this relationship. Studies on human-computer interactions emphasize the important role of emotions in technology adoption [67,68]. Emotions, as a central component of attitude toward a referent, are a mental state of readiness for action, which promote behavioral activation [67,69]. Positive emotions are responsible for the user eventually trusting the technology and using it [70].

### Limitations

The study has several limitations. First, because the survey was conducted on the internet, sampling may be biased by recruiting adults who are likely to be more technologically savvy. However, we recruited adults with SMI not only through web-based groups but also in face-to-face meetings with individuals with SMI who were interested in participating, and they completed a paper-and-pencil version of the survey. This strategy enabled us to recruit a more heterogeneous sample in terms of access to technology. Second, the majority of survey respondents were young adults with only few over the age of 65 years, which could also explain the relatively high use of technology. Therefore, the results for older adults with SMI must be interpreted with caution, and future studies should target a subgroup of older adults with SMI.

Lastly, although we acknowledge that poverty plays a crucial role in access to technology [71,72], participants in our sample enjoy the social welfare benefits provided by the In Israel Ministry of Health and the Israel National Insurance Institute. This mental health support system provides financial and rehabilitation support, including housing, education, employment, and mental health care. Therefore, although often individuals with SMI experience poverty and lack of access, respondents with SMI in our sample enjoyed social welfare benefits (Table 1), which may explain their greater access to technology. Future studies should focus on evaluating the impact of poverty on digital participation and access to technology.

### Conclusions

This study demonstrated the potential of digital community inclusion to recovery and well-being among individuals with SMI. Our findings indicate higher rates of access and use of mHealth and digital technologies among individuals with SMI than among the general population. Furthermore, our findings show that digital participation could promote recovery among adults with SMI. Accordingly, this study emphasizes the need to update and expand the definition and conceptualization of community participation, and include aspects of digital participation needs of individuals with SMI.

Our findings suggest that policy makers, service users, and researchers should use existing digital technologies and design novel mHealth interventions to support the recovery process of adults with SMI. In particular, the current COVID-19 crisis poses an opportunity for mental health care systems to adopt digital technologies for service provision. In this sense, this study, conducted before the current COVID-19 pandemic, contributes to the understating that digital participation of adults with SMI is valuable to their recovery not only in crisis but also in routine. Furthermore, it is important to support the participation of individuals with SMI in the virtual environment in a manner that facilitates a positive emotional experience. Positive emotional experience while using digital technologies is a key factor in their engagement in the web-based environment and consequently in their recovery.

## Abbreviations

mHealth: mobile health
NAMI: National Alliance on Mental Illness
SMI: serious mental illness
